# Supplementary material for: Toward Light-Responsive Hydrogel-Based Valves for Flow Regulation
Source: Langmuir. 2026 Feb 23;42(9):6702–13. doi: 10.1021/acs.langmuir.5c05520 (PMC12981012; doi:10.1021/acs.langmuir.5c05520)
Supplement: Supplementary file 1 [file la5c05520_si_001.pdf]

SUPPORTING INFORMATION

Towards light responsive hydrogel-based valves for flow regulation

Annina Mittelholzer[1#], Vincent Hickl[1,2,3#], Katharina Maniura-Weber[1], Luciano F. Boesel[2], René M. Rossi[2], Markus Rottmar[1*], Yashoda Chandorkar[1*]

[1]Empa, Swiss Federal Laboratories for Materials Science and Technology, Laboratory for Biointerfaces, CH

[2] Empa, Swiss Federal Laboratories for Materials Science and Technology, Laboratory for Biomimetic Membranes and Textiles, CH

[3] Empa, Swiss Federal Laboratories for Materials Science and Technology, Center for X-Ray Analytics, CH

*Corresponding authors

# Equal contribution

Supporting video 1:

Real-time video of confined slit valve opening under NIR illumination. For this recording, laser power was set to 95%, on-time 2s, and frequency 0.17 Hz.

Supporting video 2:

Real-time videos of an experiment described in Fig 6, showing unconfined slit valves exposed to water at 23 °C.

Supporting video 3: Real-time videos of an experiment described in Fig 6, showing unconfined slit valves exposed to water at 80 °C. Videos 2 and 3 are consecutive and show the same valve.

Supporting figures:

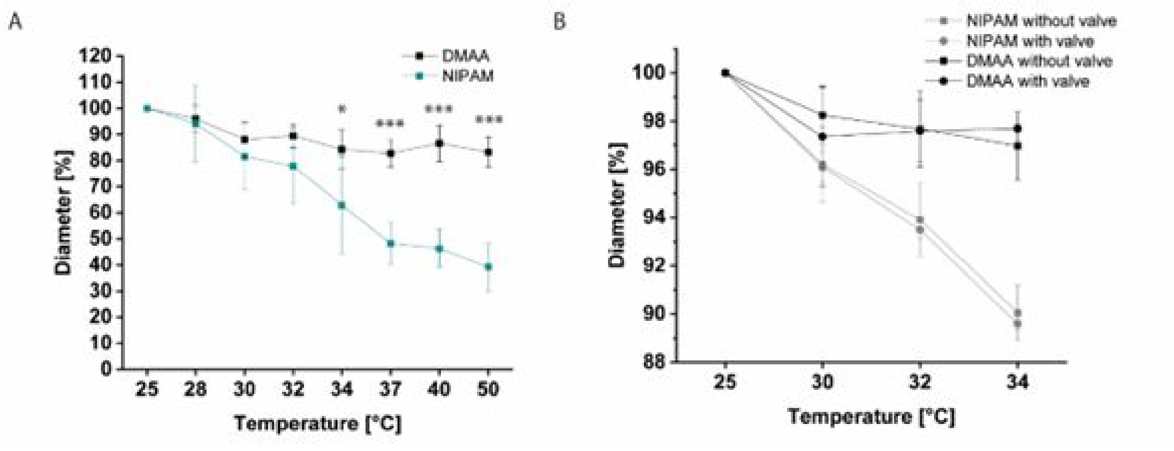

Figure S1. Experimental setup for determining the transition temperatures of PDMAA and PNIPAM hydrogels. Hydrogels (8 mm diameter) were prepared, swollen overnight, and smaller samples (n = 6) were obtained using a 3 mm biopsy punch. The samples were placed in a 48-well plate with 200 µL Milli-Q water per well. Temperature control was achieved using a customized heating mat (25–50 °C). At each temperature step, hydrogel dimensions were recorded using a NIKON camera with a 105 mm objective. Image analysis and manual diameter measurements were performed in Fiji to assess temperature-dependent swelling behavior. Thermal response of A) PDMAA and PNIPAM hydrogels showing no notable thermal response of PDMAA but a significant diameter reduction of PNIPAM with a volume phase transition temperature (VPTT) between 32 and 34 °C. indicates the significance withing the material group B) Investigation of the influence of the outlet on the diameter of PNIPAM and PDMAA hydrogels. Confined hydrogels with a 1 mm hole outlet (n = 3) and without an outlet (n = 3) were placed in a 30 mm Petri dish containing 3 mL of Milli-Q water. Diameters were measured at 25, 30, 32, and 34 °C. For imaging, hydrogels were briefly transferred into a 60 mm Petri dish without Milli-Q water to improve visualization, particularly of PDMAA samples, before being returned to the original dish to prevent dehydration. Manual diameter measurements were performed using Fiji. Statistical analysis revealed no significant differences between samples with and without an outlet, indicating that the presence of the outlet does not affect the diameter of PNIPAM or PDMAA hydrogels. confined hydrogels (PNIPAM and PDMAA measured) without and with valve. *, **, *** were determined using one-way ANOVA and are statistically significant at $p < 0.05$, 0.01 and 0.001, respectively.*

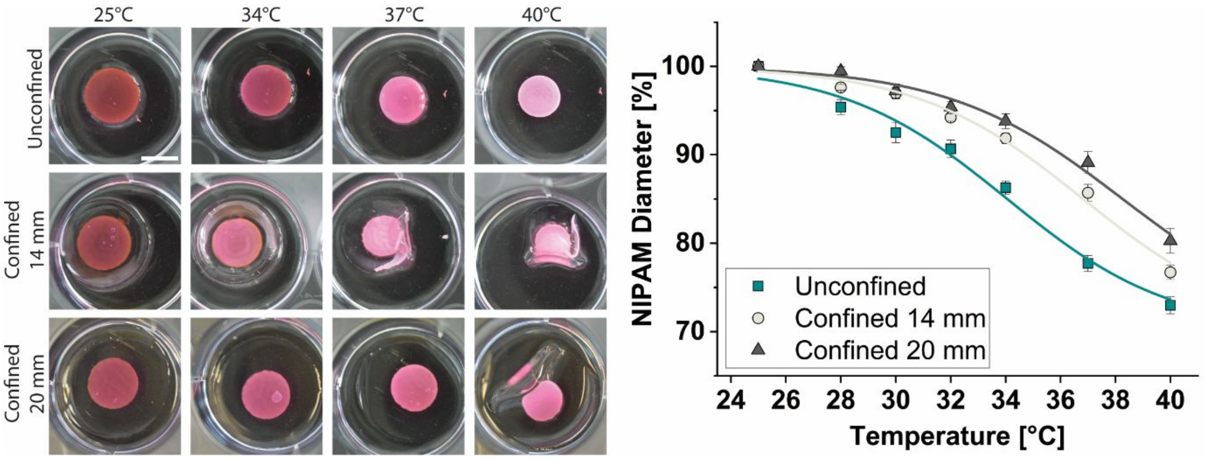

Figure S2. Left: Photos showing the unconfined, and confined gels (without outlet), with 2 different degrees of confinement (14 mm and 20 mm). Scale bar 1 cm. Right: Diameter of unconfined and confined hydrogels as a function of temperature (reproduced from Fig 2). Solid lines represent logistic fits according to the equation $D = A - \frac{B}{1+e^{-k(T-T_0)}}$ *with fitting parameters shown in Table S1.*

	**A**	**B**	**k**	**T_0**	**Adjusted R^2**
**Unconfined**	100	30	0.334	34.06	0.98
**Confined 14 mm**	100	30	0.330	36.82	0.99
**Confined 20 mm**	100	30	0.314	38.26	0.98

Table S1. Fitting parameters for logistic fits to PNIPAM diameters as functions of temperature (see Fig S2). Parameters A and B were chosen manually and fixed to allow the fits to converge and to avoid overfitting. The remaining parameters k and T_0 describe the overall rate of change of the diameter near the transition and the temperature at which the rate of change appears to be highest, respectively.

Two-way ANOVA					
Alpha	0.05				
**Source of Variation**	**% of total variation**	**P value**	**P value summary**	**Significant?**	
Interaction	2.906	<0.0001	****	Yes	
Temperature	84.18	<0.0001	****	Yes	
Confinement	8.248	<0.0001	****	Yes	
**ANOVA table**	**SS**	**DF**	**MS**	**F (DFn, DFd)**	**P value**
Interaction	250	12	20.84	F (12, 105) = 5.454	P<0.0001
Temperature	7244	6	1207	F (6, 105) = 316.0	P<0.0001
Confinement	709.7	2	354.9	F (2, 105) = 92.89	P<0.0001
Residual	401.1	105	3.82		
**Data summary**					
Columns (Confinement)	3				
Rows (Temperature)	7				
Number of values	126				

Table S2. Two-way ANOVA results for hydrogel disk diameters as functions of both temperature and confinement (data shown in Fig 2 and Fig S2). Analysis performed using GraphPad Prism.

Tukey's multiple comparisons test	Mean diff.	95.00% CI of diff.	Summary	Adjusted P Value
**25 °C**				
Unconfined vs. Confined 14 mm	0	-2.173 to 2.173	ns	>0.9999
Unconfined vs. Confined 20 mm	0	-2.173 to 2.173	ns	>0.9999
Confined 14 mm vs. Confined 20 mm	0	-2.173 to 2.173	ns	>0.9999
**28 °C**				
Unconfined vs. Confined 14 mm	-2.235	-4.918 to 0.4475	ns	0.1220
Unconfined vs. Confined 20 mm	-4.070	-6.753 to -1.388	**	0.0014
Confined 14 mm vs. Confined 20 mm	-1.835	-4.518 to 0.8478	ns	0.2392
**30 °C**				
Unconfined vs. Confined 14 mm	-4.430	-7.113 to -1.748	***	0.0005
Unconfined vs. Confined 20 mm	-4.687	-7.370 to -2.005	***	0.0002
Confined 14 mm vs. Confined 20 mm	-0.2570	-2.940 to 2.426	ns	0.9718
**32 °C**				
Unconfined vs. Confined 14 mm	-3.565	-6.248 to -0.8826	**	0.0058
Unconfined vs. Confined 20 mm	-4.746	-7.428 to -2.063	***	0.0002
Confined 14 mm vs. Confined 20 mm	-1.180	-3.863 to 1.503	ns	0.5498
**34 °C**				
Unconfined vs. Confined 14 mm	-5.602	-8.285 to -2.920	****	<0.0001
Unconfined vs. Confined 20 mm	-7.514	-10.20 to -4.831	****	<0.0001
Confined 14 mm vs. Confined 20 mm	-1.912	-4.595 to 0.7709	ns	0.2122
**37 °C**				
Unconfined vs. Confined 14 mm	-7.987	-10.67 to -5.304	****	<0.0001
Unconfined vs. Confined 20 mm	-11.39	-14.07 to -8.707	****	<0.0001
Confined 14 mm vs. Confined 20 mm	-3.402	-6.085 to -0.7196	**	0.0090
**40 °C**				
Unconfined vs. Confined 14 mm	-3.732	-6.415 to -1.049	**	0.0037
Unconfined vs. Confined 20 mm	-7.305	-9.988 to -4.622	****	<0.0001
Confined 14 mm vs. Confined 20 mm	-3.573	-6.256 to -0.8900	**	0.0057

Table S3. Results of post-hoc test following the ANOVA in Table S2. Tukey multiple comparisons tests were performed to test whether the degree of confinement was significant at each temperature. Multiplicity-adjusted p-values are reported to account for multiple comparisons.

Two-way ANOVA	Ordinary
Alpha	0.05

Source of Variation	% of total variation	P value	P value summary	Significant?
**Circular valve**				
Interaction	11.14	<0.0001	****	Yes
Temperature	53.71	<0.0001	****	Yes
Confinement	13.35	<0.0001	****	Yes
**Slit valve**				
Interaction	6.727	0.0183	*	Yes
Temperature	49.69	<0.0001	****	Yes
Confinement	3.580	0.0068	**	Yes

ANOVA table	SS (Type III)	DF	MS	F (DFn, DFd)	P value
**Circular valve**					
Interaction	2260	5	451.9	F (5, 86) = 25.53	P<0.0001
Temperature	10899	5	2180	F (5, 86) = 123.2	P<0.0001
Confinement	2709	1	2709	F (1, 86) = 153.1	P<0.0001
Residual	1522	86	17.70		
**Slit valve**					
Interaction	593690	5	118738	F (5, 90) = 2.887	P=0.0183
Temperature	4385755	5	877151	F (5, 90) = 21.33	P<0.0001
Confinement	315968	1	315968	F (1, 90) = 7.683	P=0.0068
Residual	3701227	90	41125		

Difference between column means	Circular valve	Slit valve
Predicted (LS) mean of Unconfined	81.29	219.1
Predicted (LS) mean of Confined	91.95	330.6
Difference between predicted means	-10.66	-111.5
SE of difference	0.8615	40.23
95% CI of difference	-12.37 to -8.946	-191.4 to -31.59

Data summary	Circular valve	Slit valve
Number of columns (Confinement)	2	2
Number of rows (Temperature)	6	6
Number of values	98	102

Table S4. Two-way ANOVA results for valve outlet areas as functions of both temperature and confinement (data shown in Fig 3). Values for circular and slit valve are shaded blue and green, respectively. Analysis performed using GraphPad Prism.

Tukey's multiple comparisons test	Predicted (LS) Mean diff.	95.00% CI of diff.	Summary	Adjusted P Value
Unconfined vs. Confined				
**Circular valve**				
25	0.000	-4.064 to 4.064	ns	>0.9999
28	-0.8489	-4.913 to 3.215	ns	0.6790
30	-5.332	-9.396 to -1.268	*	0.0107
32	-11.07	-15.13 to -7.007	****	<0.0001
34	-17.84	-22.06 to -13.63	****	<0.0001
37	-28.86	-33.52 to -24.19	****	<0.0001
**Slit valve**				
25	0.000	-195.8 to 195.8	ns	>0.9999
28	3.200	-192.6 to 199.0	ns	0.9742
30	-8.993	-204.8 to 186.8	ns	0.9275
32	-49.77	-245.5 to 146.0	ns	0.6147
34	-197.1	-392.9 to -1.355	*	0.0485
37	-416.4	-612.1 to -220.6	****	<0.0001

Table S5. Results of post-hoc test following the ANOVA in Table S4. Tukey multiple comparisons tests were performed to test whether the degree of confinement was significant at each temperature. Multiplicity-adjusted p-values are reported to account for multiple comparisons. Values for circular and slit valve are shaded blue and green, respectively.

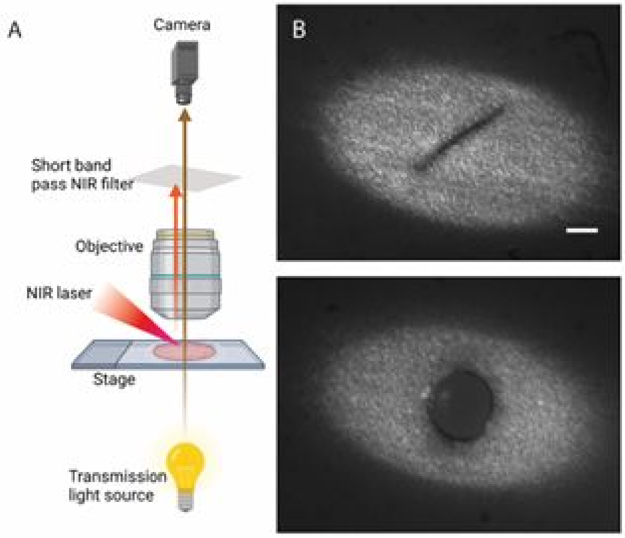

Figure S3. (A) The hydrogel sample is irradiated with near-infrared (NIR) light (shown in orange) directed through the microscope objective. A short-pass NIR filter is placed in the detection path to protect the camera by blocking NIR wavelengths while allowing visible light (brown) to pass through. (B) The images show the centralized alignment of the laser spot on the outlet. The laser spot size (~1.5 mm²) is sufficiently large to cover the entire outlet area, ensuring uniform irradiation across the hole. Scale bar 200 µm

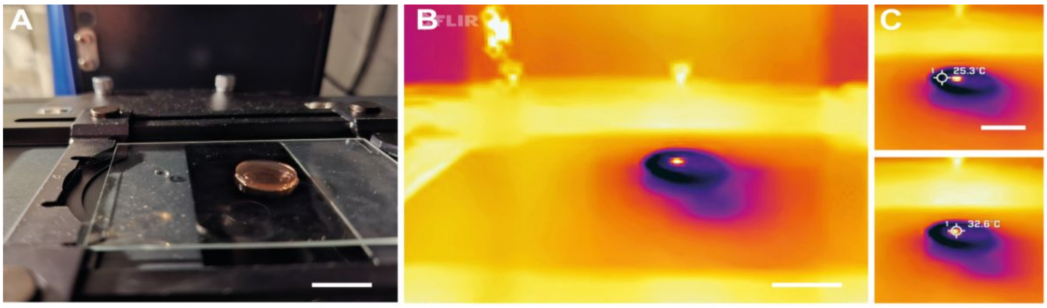

Figure S4. (A) Unconfined (PNIPAM) hydrogel was located under the microscope as a reference. Thermal imaging was done with the FLIR One Edge Pro. (B) The laser, with a power of 153 mW, pulsed on the unconfined PNIPAM hydrogel for three cycles (on time 1'000 ms, interval 20'000 ms). The color map iron sheet (Eisenblech) was used for all images. The video of the three cycles running was taken (B) without temperature measurement and with temperature measurement in the (C) center of the laser spot (32.6 °C) as well as the periphery of the PNIPAM hydrogel (25.3 °C). The IR images showing here no temperature change in the periphery but a centralized heating that is local around the laser spot. Scale bar 1 cm.

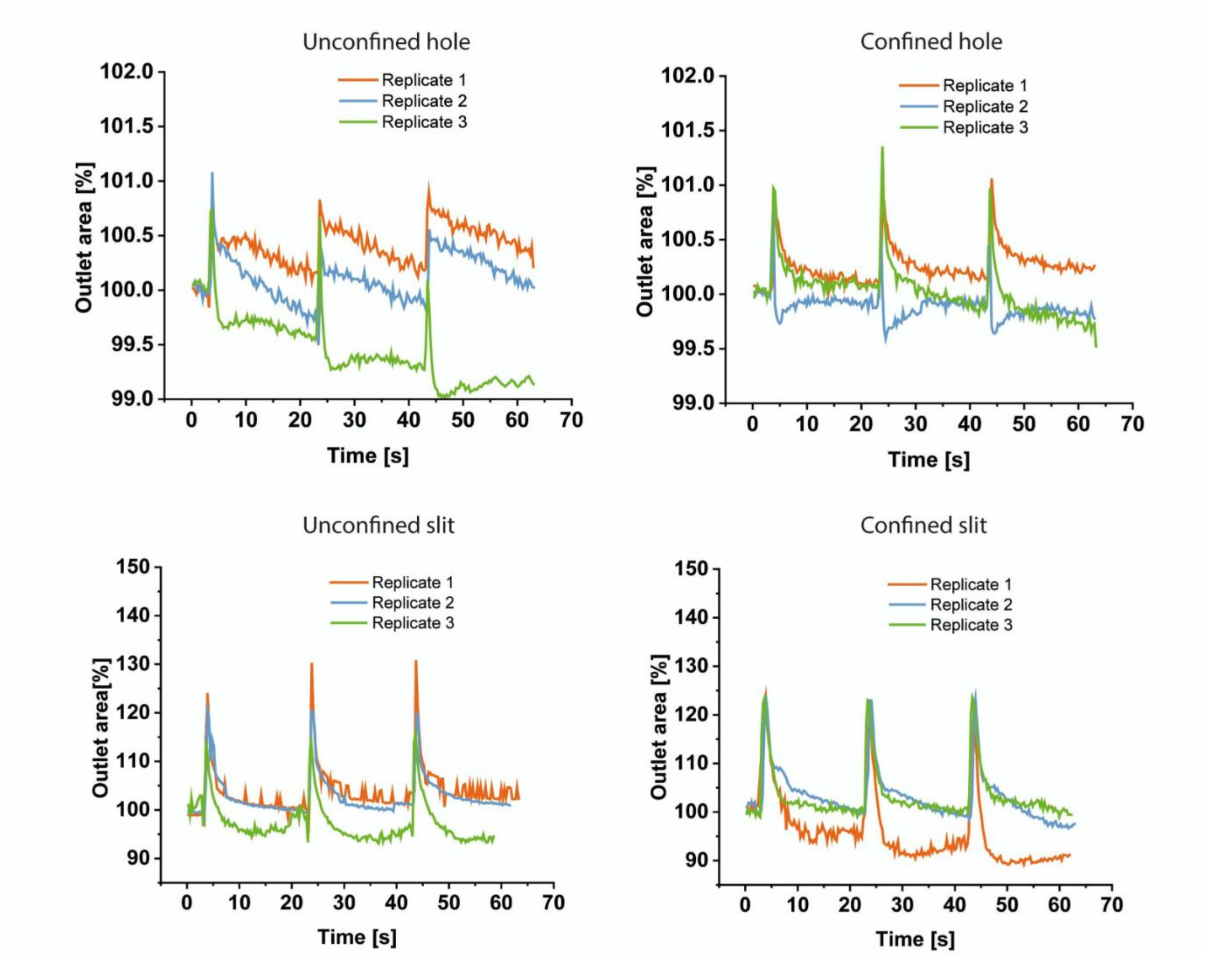

Figure S5. Replicates of the valves tested as described in Fig 5: unconfined hole, confined hole, unconfined slit and confined slit.

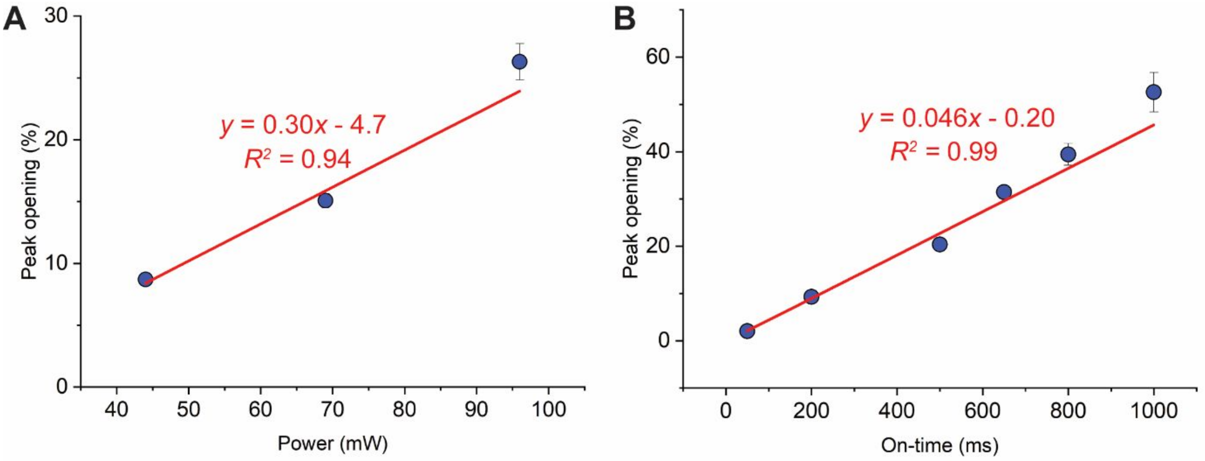

Figure S6. Effect of changing (A) laser power and (B) on-time on opening of the slit valve. Peak openings (%) are the difference between the maximum size of the valve outlet immediately after a laser pulse and the previous minimum size immediately before the pulse. Each data point in (A) and (B) is an average taken across the 5 or 3 pulses shown in Fig 6A and 6B, respectively. Error bars represent standard errors. Red lines represent linear best fits, with fit parameters and adjusted R^2 values shown.

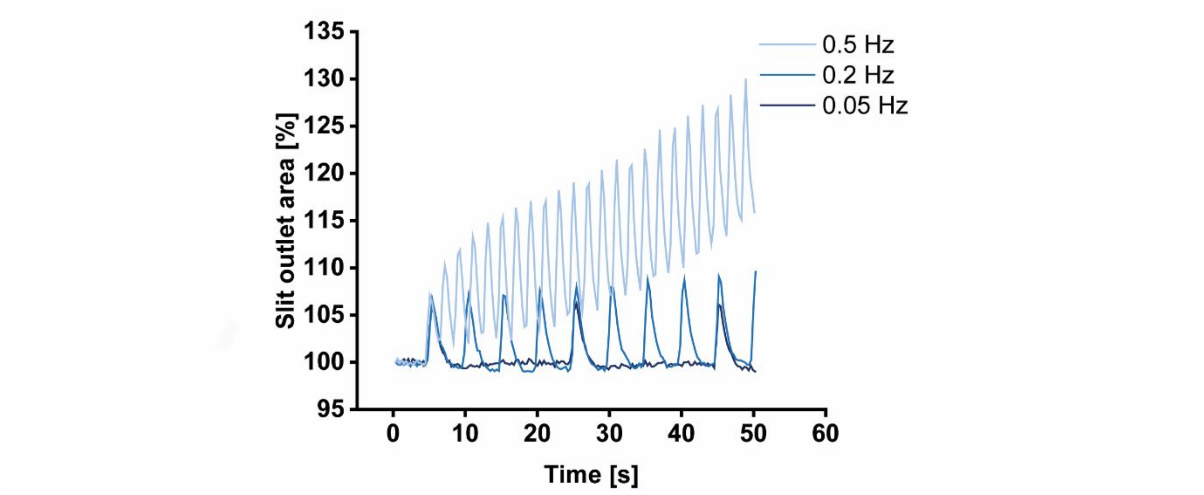

Figure S7. Effect of changing laser pulse frequency on change in area of the slit outlet. The pulse frequency was varied at constant laser power (96 mW) and pulse duration (500 ms).

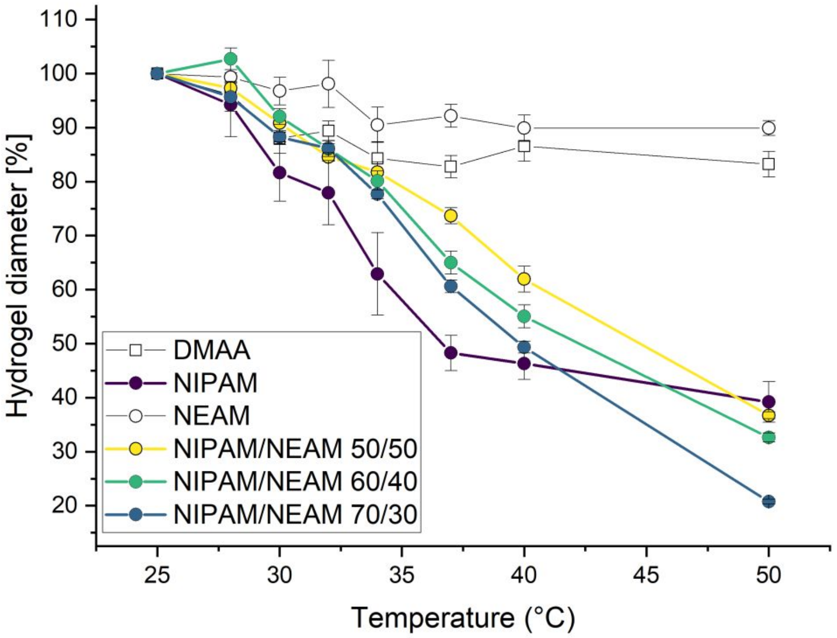

Figure S8. Diameter of different hydrogels as a function of temperature, measured as a percentage of each gel's diameter at 25 °C. The negative controls of PDMAA and PNEAM do not change size significantly. Compared to pure NIPAM, copolymers of NIPAM and NEAM have a higher volume phase transition temperature (VPTT).

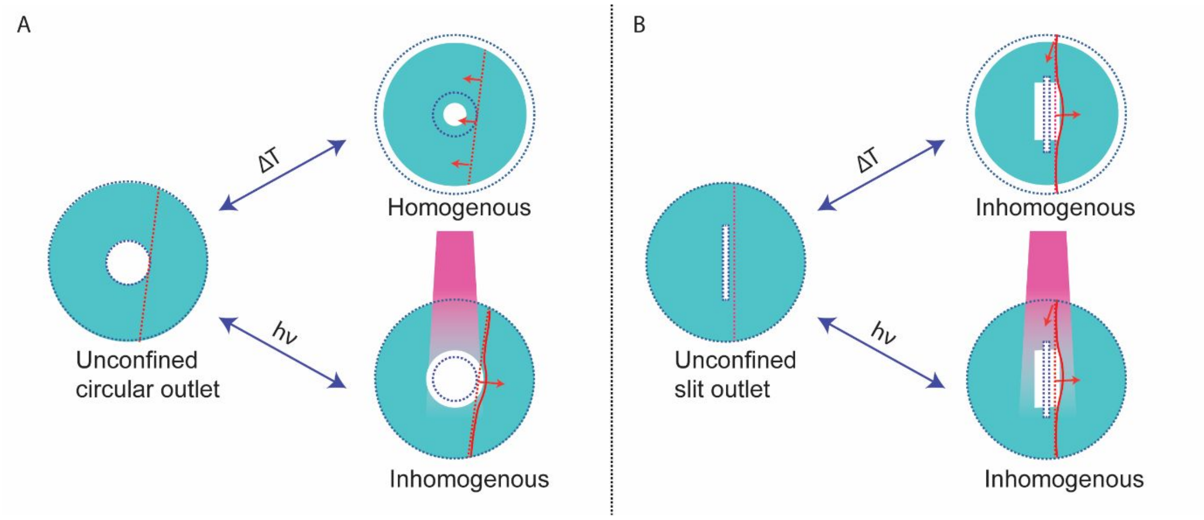

Figure S9. Changes in temperature or light affect the deformation of two types of outlets in the unconfined hydrogel: (A) circular outlet and (B) slit outlet. These changes result in homogenous or inhomogeneous deformation. Dotted line shows original shape of the outlet.

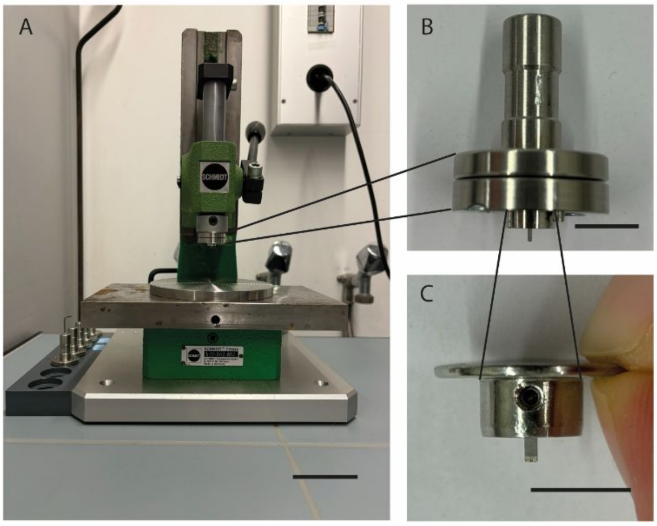

Figure S10. (A) The mechanical press (scale 5 cm) with (B) the clickable plugin (scale 1 cm) with (C) the beaver blade holder (scale 1 cm), which acts as tongs for the 1 mm beaver blade.

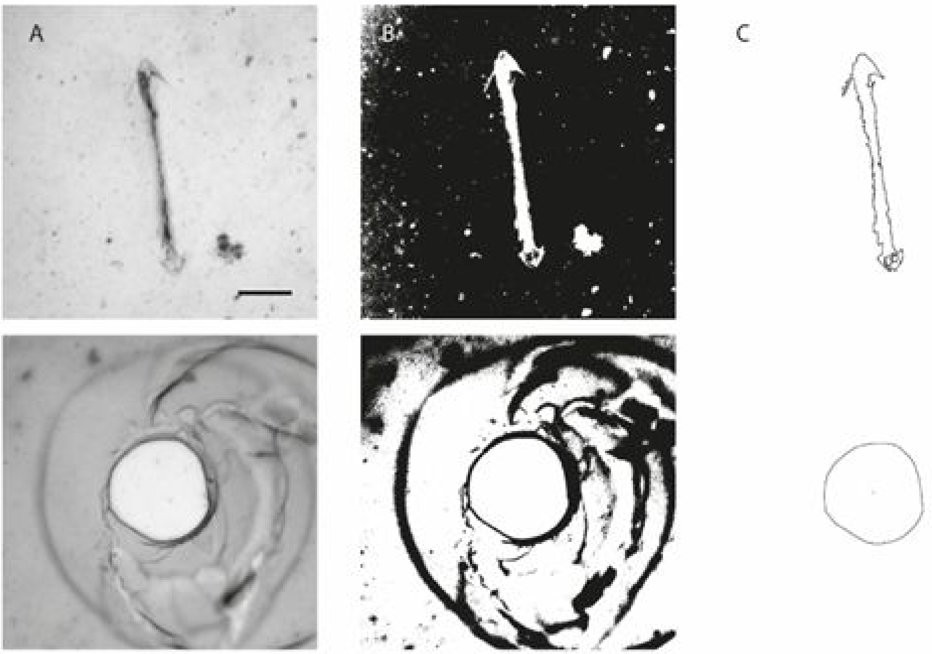

*Figure S11. Evaluation sequence of the outlets containing the (A) original image, (B) auto thresholding using Huang (temperature) or Otsu (light) and (C) drawing of the outlet area. *slit is here only for the light measurement, in the temperature measurement it was measured manually (Fig S12) Scale bar 200 µm.*

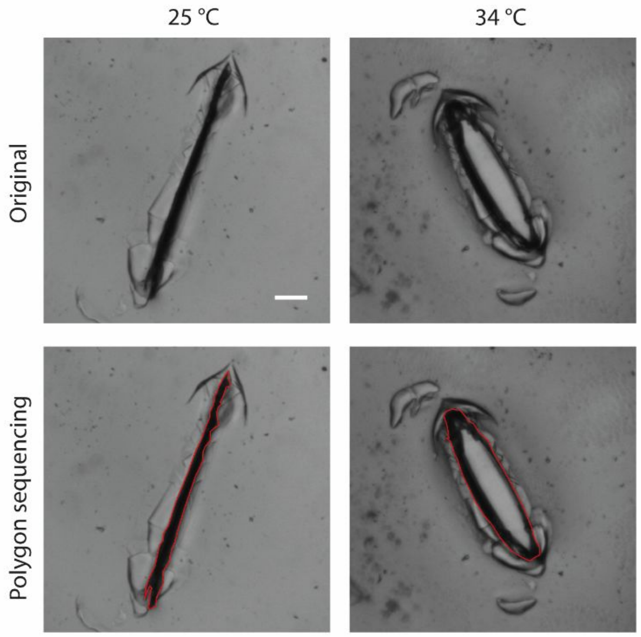

Figure S12. Example for the slit area measurement in the temperature tests where first the original slit image is shown and then the evaluation of the outlet area using polygon sequencing (by hand) in Fiji where the outermost slit area is being measured.

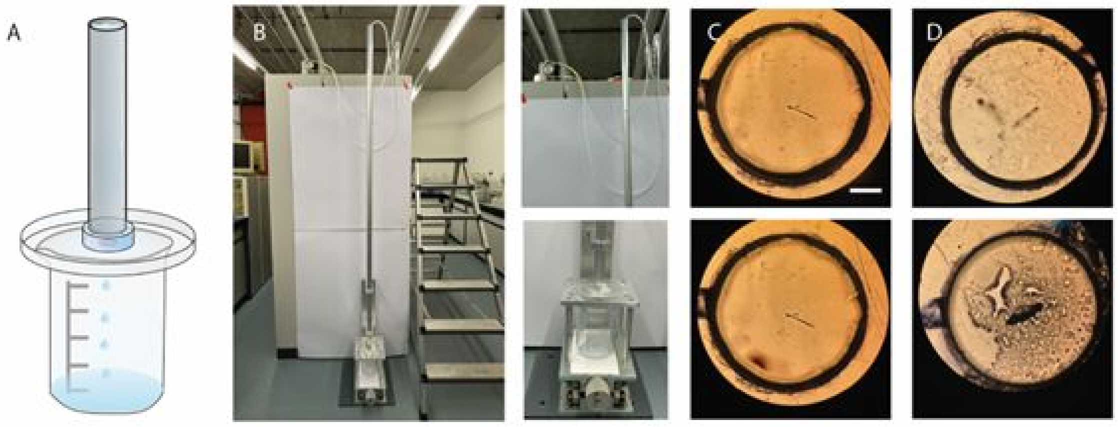

Figure S13. (A) Schematic of the pressure and flow measurement setup, where the hydrogel is placed on a petri dish with a 4 mm hole and an aluminum rod is tightly aligned with the hydrogel. Between the hydrogel and the aluminum rod, a PDMS ring is placed to prevent the hydrogel from breaking. This setup allows for the simulation of the pressure using hydrostatic pressure. (B) Pressure setup with tubing connected to a pump, enabling the controlled filling of the aluminum rod (I.D.= 2 cm, l =2 m) and a height-adjustable table ensuring a tight seal between hydrogel and rod interface. Position of the slit outlet on the hole of the petri dish before and after (C) pressure measurement and (D) flow measurement. Scale bar 1 mm

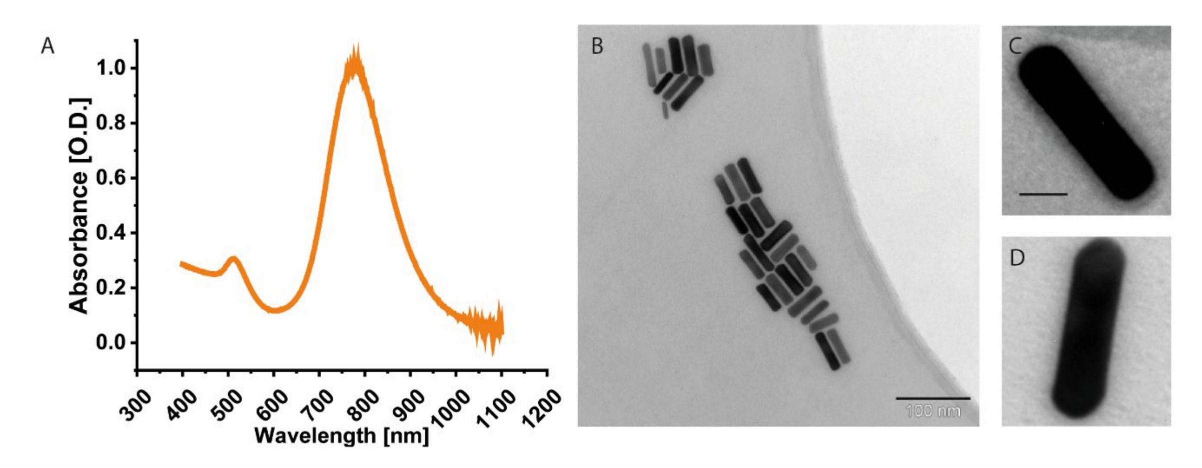

Figure S14 (A) AuNRs in DMSO with an absorbance peak at 790 nm were produced, which matches the wavelength of the NIR laser at 800 nm. Since no shift of the peak is observed, any aggregation of the NRs is considered negligible. (B) TEM images showing a AuNRs A.R. of 3.7 and the nanorod dimensions of 49 nm in length and 13 nm in width. Scale bar 100 nm. (C) The pegylated (indicated by the dark shadow around the AuNRs) and (D) non-pegylated AuNRs. scale bar 20 nm.

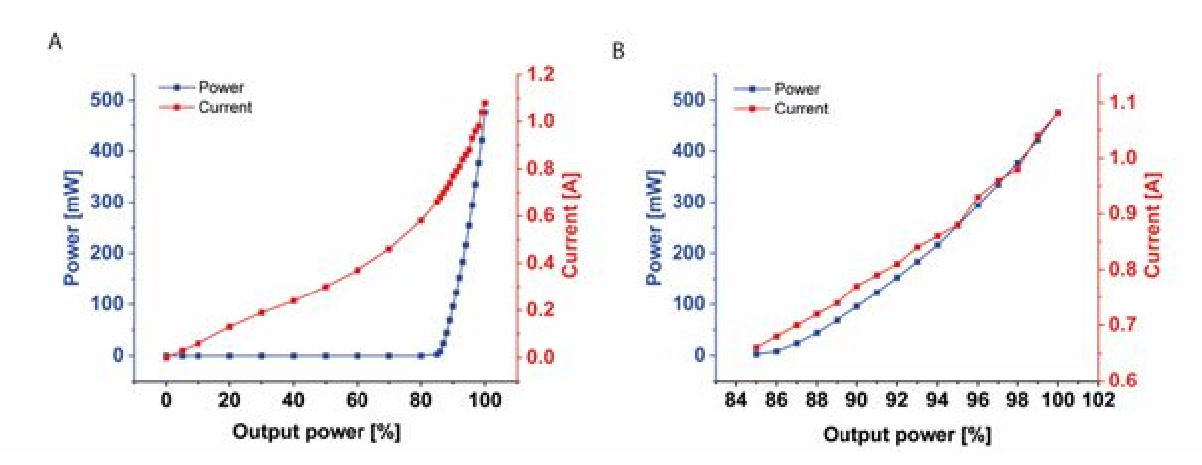

Figure S15. The laser power output through the adjustable collimator was measured after reducing the laser power at the laser head aperture to 480 mW. A PM2 sensor (2 W, Coherent) in combination with a FieldMax II power meter (Coherent) was used for this measurement. To ensure consistent results, the distance between the collimator and the sensor, as well as the collimator's focus, were kept constant throughout the experiment. (A) Laser power settings below 85% showing that the measured output power remained zero, despite a continuous increase in current. Beyond this threshold, both the output power and current increased continuously. (B) The detailed behavior of the system above 85% laser power, where a nearly linear relationship of power and current is observed. At 100% laser power, the system operated at a current of 1.1 A with a measured output power of 476 mW.
